# Characterization of Dual-Layer Hybrid Biomatrix for Future Use in Cutaneous Wound Healing

**DOI:** 10.3390/ma16031162

**Published:** 2023-01-29

**Authors:** Izzat Zulkiflee, Ibrahim N. Amirrah, Nur Izzah Md Fadilah, M. F. Mohd Razip Wee, Salma Mohamad Yusop, Manira Maarof, Mh Busra Fauzi

**Affiliations:** 1 Centre for Tissue Engineering and Regenerative Medicine, Faculty of Medicine, Universiti Kebangsaan Malaysia, Jalan Yaakob Latiff, Bandar Tun Razak, Cheras, Kuala Lumpur 56000, Malaysia; 2 Institute of Microengineering and Nanoelectrics, Universiti Kebangsaan Malaysia, Bangi 43600, Malaysia; 3 Department of Food Sciences, Faculty of Science and Technology, Universiti Kebangsaan Malaysia, Bangi 43600, Malaysia

**Keywords:** collagen, gelatin, PVA, bilayer scaffold, elastin, wound healing

## Abstract

A skin wound without immediate treatment could delay wound healing and may lead to death after severe infection (sepsis). Any interruption or inappropriate normal wound healing, mainly in these wounds, commonly resulted in prolonged and excessive skin contraction. Contraction is a common mechanism in wound healing phases and contributes 40–80% of the original wound size post-healing. Even though it is essential to accelerate wound healing, it also simultaneously limits movement, mainly in the joint area. In the worst-case scenario, prolonged contraction could lead to disfigurement and loss of tissue function. This study aimed to fabricate and characterise the elastin-fortified gelatin/polyvinyl alcohol (PVA) film layered on top of a collagen sponge as a bilayer hybrid biomatrix. Briefly, the combination of halal-based gelatin (4% (*w*/*v*)) and PVA ((4% (*w*/*v*)) was used to fabricate composite film, followed by the integration of poultry elastin (0.25 mg/mL) and 0.1% (*w*/*v*) genipin crosslinking. Furthermore, further analysis was conducted on the composite bilayer biomatrix’s physicochemical and mechanical strength. The bilayer biomatrix demonstrated a slow biodegradation rate (0.374967 ± 0.031 mg/h), adequate water absorption (1078.734 ± 42.33%), reasonable water vapour transmission rate (WVTR) (724.6467 ± 70.69 g/m^2^ h) and porous (102.5944 ± 28.21%). The bilayer biomatrix also exhibited an excellent crosslinking degree and was mechanically robust. Besides, the elastin releasing study presented an acceptable rate post-integration with hybrid biomatrix. Therefore, the ready-to-use bilayer biomatrix will benefit therapeutic effects as an alternative treatment for future diabetic skin wound management.

## 1. Introduction

In tissue engineering, biomatrices are very common as they provide desirable growth conditions for cells that mimic our bodies. A biomatrix is usually a porous three-dimensional (3D) scaffold that provides an appropriate surrounding microenvironment, especially for regenerating tissues and organs [1,2,3]. Each 3D-scaffold should provide properties as such: architecture and mechanical strength, composition of the biomaterial, which will determine the cellular interaction’s efficacy, functions in tissue regeneration along with effective host tissues integration [4,5]. Thus, biomaterials from synthetic and natural polymers have been widely explored for biomatrix fabrication to satisfy these properties. On another note, one of the naturally occurring polymers utilized often in numerous industries is gelatin, especially in clinical settings and bioengineering applications. Gelatin is a highly compatible, highly soluble, abundant and cheap raw material and, most importantly, biodegradable [6,7,8]. Gelatin is a collagen derivative through different approaches: heating or enzymatic, to produce authentic short peptides. It shows less toxicity towards cells [9,10,11,12] as its existing properties were not far from native collagen, which is widely abundant in human skin. It has very low immunogenicity and can be integrated into various tissues. However, it has relatively low mechanical properties that can limit its function for the applications of tissue engineering. Despite that, a study by Amadori et al., 2015 [13] exhibited that gelatin was recommended to have better biological performance with a crosslinking agent such as genipin.

Next, genipin obtained from gardenia fruit (Gardenia jasminoides ELLIS) is a natural crosslinking agent. Using a modern microbiological technique, genipin, a bi-functional water-soluble crosslinking reagent, is isolated from gardenia fruits [14]. It quickly reacts with chitosan and proteins to create fluorescent hydrogels that are blue in hue [15]. In addition, it provides mechanical characteristics, tensile strength and thermal stability towards gelatin [16]. Another alternative to improve these materials’ mechanical properties could be adding these biopolymers to synthetic monomers or polymers. PVA, a synthetic polymer which is Food and Drug Administration (FDA) approved, can be used as an alternative as it is polar and water-soluble, and it is also acknowledged as one of the biodegradable synthetic polymers under anaerobic and aerobic environments [17,18]. There are many treatments and research going on towards gelatin to improve and modify the properties of gelatin to help in developing new materials combining polymers from synthetic and natural polymers for desired properties. In the future, newly combined bio-artificial polymeric materials’ bioscaffolds, such as films, could be used and applied in many fields, especially medical ones. Collagen has been a structural protein widely used in tissue engineering for the last ten years because it supports cell growth and has promoted tissue repair. It is an enormous protein content in the human body and can be found in connective tissues such as the skin, tendons and bones. It is a good option for the application in the body, either produced synthetically or taken from natural sources aside from being biocompatible and biodegradable. Numerous tissue engineering applications, including bone regeneration, wound healing and drug delivery, have used collagen as a fundamental biomaterial [19,20]. The ability of collagen to be altered or functionalized to improve its characteristics and applicability for various applications is a crucial component in tissue engineering. For instance, collagen can be chemically altered to make it more stable or functionalized, with biomolecules such as peptides or enzymes to support biological functions. These changes may help collagen fulfil its potential as a biomaterial for regenerative medicine and tissue engineering applications.

Moreover, elastin can be found primarily in various tissues in fibre form, providing resilience and elasticity in skin, arteries, ligaments, lungs and others [21]. Elastin was expressed in the extracellular matrix (ECM) after the first few years of life, specifically in the late and early neonatal periods [22]. In a sense, the elastin expression in our body decreases once we grow older. Elastin fibres, formed starting with tropoelastin (the precursor of elastin), experienced a coacervation process to form spherules and were secreted out into the extracellular matrix after being expressed by elastogenic cells such as smooth muscle cells and fibroblasts [23]. The spherules are then deposited onto microfibrils forming elastin fibres [24]. As one of the most stable proteins, with a half-life of around 74 years, elastin is crucial for the healthy operation of elastic connective tissues. In addition, the hydrophobic and cross-linked nature of elastin makes it resistant to enzymatic proteolysis. However, elastin degradation by fibroblast elastases (MMP, HLE, CG) is related to the progression of several diseases affecting multiple organs and tissues [21]. Outsourced elastin can be obtained by extracting it from animals. Waste products such as poultry skin can be processed to extract elastin, instead of being wasted. Numerous products, including anti-aging cosmetics, medications and tissue regeneration regimens, can benefit from using elastin [25,26]. The application of elastin incorporated into the scaffold is commonly used in the medical field; however, there are few studies emphasizing elastin as the main bioactive in biomatrix as a potential therapeutic approach.

**Figure 1:**
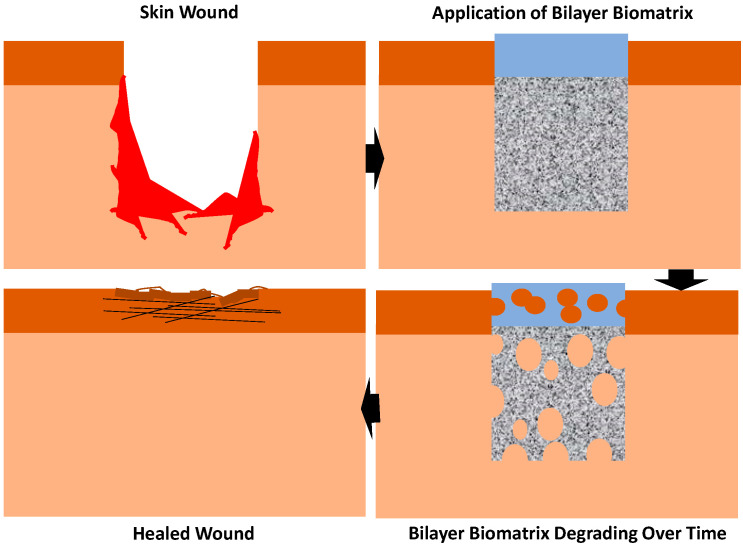
Application of bilayer biomatrix on a skin wound.

Wound healing is an automatic series of coordinated events, like a cascade, involving complex physiological processes that are very important in our body. Skin injuries often occur from accidents or burning that lead to skin wound healing. There are four overlapping dynamic stages of wound healing, and these include haemostasis, inflammation and the proliferative and remodelling stage [27]. Haemostasis is essentially the initial stage of healing where blood clots are formed to halt the bleeding. Inflammation is where neutrophils destroy bacteria, remove debris and prepare for the wound bed to grow new tissues. The proliferative phase is where it fills and covers the wounds and skin contraction occurs. Finally, the new tissues slowly gain strength and flexibility in the remodelling phase. During the proliferation phase, myofibroblasts are crucial for the contraction of the wound [28]. Due to the wound closing, the contraction of a skin wound is one of the crucial stages in wound healing [29,30]. Myofibroblasts, however, provide a situation that is similar to a double-edged sword, because it is advantageous on one side since it helps to narrow the defect, but on the other side, a severe action can result in unfavourable and unsightly scarring [31]. Diabetic wounds commonly happen in diabetic patients and usually lead to amputations due to the lack of proper wound care. This wound developed because diabetes damages the nerves and blood vessels in the feet. A chronic wound such as this is very slow healing and prone to infection and typically leads to severe consequences, such as losing a limb or multiple amputations. Figure 1 shows the application of the bilayer biomatrix on a skin defect. 

This study aimed to fabricate and characterize the physicochemical and mechanical properties of the bilayer bioscaffold with the purpose of wound healing treatments that are made up from high grade halal-based gelatin with PVA and combined with elastin from poultry skin to form a film, layered on top of a collagen sponge. After that, it is followed by the evaluation of cytotoxicity effects between elastin and human dermal fibroblasts, as well as the released of elastin through biodegradation using elastin assay. This bilayer bioscaffold is a suitable acellular natural 3D scaffold that can mimic the extracellular matrix of the skin as well as providing rapid treatments without adding any drugs or formulation for wound healing treatment. Adding drugs or a formulation often complicates matters such as stability, homogeneity, uniformity and segregations [32]. Instead, the usage of promising natural sources’ biomaterials can benefit more without considering the side effects of the drugs or the potential for the drug to not work as intended. Using acellular natural source scaffolds has much potential and can be highly suitable for wound healing treatments [33,34,35,36,37].

## 2. Materials and Methods

All relevant protocols were approved for this study by the Universiti Kebangsaan Malaysia (UKM) Research Ethics Committee (UKM PPI/111/8/JEP-2019-677).

### 2.1. Fabrication of Collagen Sponge

The ovine tendon was extracted into a collagen solution from blending sheep legs and was soaked in 0.35 M (*v*/*v*) acetic acid (Merck, Darmstadt, Germany) overnight (12 h) at 4 °C. This collagen extraction procedure was published by Fauzi et al., (2016) [34,38] to obtain collagen type I, then salting out with sodium chloride (NaCl; Merck, Darmstadt, Germany) and dialyzed. Finally, pre-freezing of the collagen solution at −80 °C was carried out before the freeze-drying procedure. At concentration 15 mg/mL, the dried collagen was weighed and dissolved in 0.35 M (*v*/*v*) at 4 °C. The collagen solution was pre-frozen in a desired mould at −80 °C for 6 h and freeze-dried (Ilshin, Gyeonggido, Republic of Korea) for 24 to 48 h.

### 2.2. Fabrication of Hybrid Film Forming the Dual-Layer Scaffold

**Figure 2:**
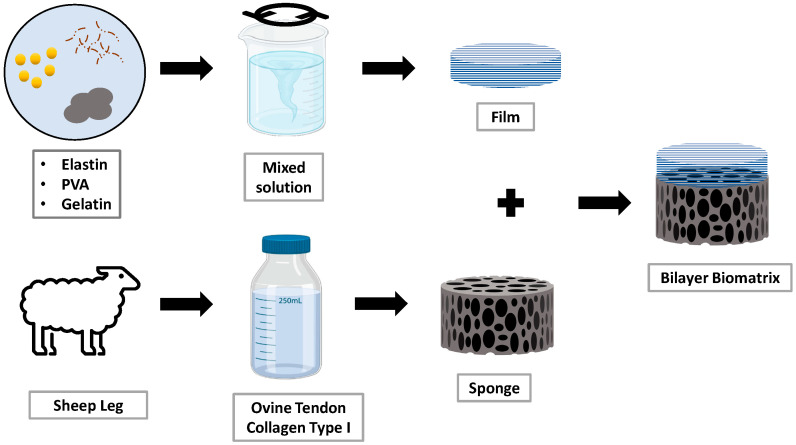
Workflow of fabrication of the bilayer biomatrix.

Gelatin (Nitta-Gelatin) and polyvinyl alcohol (PVA) powder (Mw 70,000 g/mol, partially hydrolysed ≥85%, MERCK KGaA, Darmstadt, Germany) were mixed and dissolved together by adding the powders into water with constant stirring. Gelatin containing PVA solution was blended for preparing films. The solutions were blended in hot water around 40–50 °C for about 90–100 min to produce a homogeneous solution. After that, the solution was added with elastin and was then added with genipin (0.1%; *w*/*v*). The final solution was then casted onto a silicon mould to form a film. The solutions were maintained in a thickness of 6 mm on the mould. As it semi-polymerized, forming a hydrogel after a few minutes, the crosslinked collagen sponge was placed on top of the semi-polymerized hydrogel and allowed to dry for few days. The bilayer was fabricated after the hydrogel part of the bilayer completely dried and was kept in the chiller at 4 °C. Figure 2 shows the method of fabrication of the bilayer biomatrix.

### 2.3. Swelling Study

The study for swelling properties of the bilayer scaffold was calculated using the method from a previous study by Khoushabi et al. [39] with some minor modifications. In brief, the initial weight (dry form) of the bioscaffold was measured first, and then the bioscaffold was soaked in phosphate-buffered saline (PBS) at room temperature at different periods. The swelling ratio (SR) was analysed using the following formula:

SR = [Wf − Wi]/Wi × 100,

where Wi and Wf are the weight of the scaffold in dry and hydrated forms, respectively.

### 2.4. In Vitro Biodegradation

A 0.0006% collagenase type I solution was used to measure the biodegradation rate through enzymatic degradation. After being weighted, the biocomposite scaffold was submerged in 0.0006% collagenase, incubated at 37 °C. Using the following formula, the rate of biodegradation (BP) was calculated:

BP = [Wi − Wf]/Time,

where Wi is the initial weight and Wf is the final weight.

### 2.5. Concentration of Amine Group

The concentration of the amine group was discovered using ninhydrin assay (Sigma Aldrich, Saint Louis, MO, USA). The proper standard curve was prepared by a serial dilution (0.06, 0.125, 0.25, 0.5 and 1 mg/mL) using glycine. The bioscaffolds were then weighed at 10 mg of the scaffold immersed into 200 µL of 10% ninhydrin solution. The bioscaffolds were boiled at 100 °C for 2 min and cooled down before diluting the solution by adding 95% of ethanol. A 100 µL portion of the solution was transferred into a 96-well plate and analysed using a spectrophotometer (BioTek, PowerWave XS, Highland Park, Winooski, VT, USA) with absorbance at 570 nm. The concentration of the amine group was determined using the formula below:

Concentration of amine group = [As − Yint]/Gradient]

where Anc is absorbance scaffold, Yint is the Y-intercept and Gradient is the gradient of the straight line of the standard curve.

### 2.6. Water Vapour Transmission Rate

The water vapour transmission rate (WVTR) was evaluated according to the American Society for Testing and Materials (ASTM) standard and was adapted from Rui et al., 2016 [40]. Ten mL of distilled water was prepared in a glass bottle and the bioscaffold was layered on top, closing the surface cap of the bottle. The sample was placed in an incubator in a controlled environment (5% CO_2_ and 37 °C) for 24 h. The results were recorded and analysed using the formula below:

WVTR = (Wi − Wf)/(A × Time),

where Wi: initial weight, Wf: final weight and A: surface area of cylinder bottle, respectively.

### 2.7. Simple Mechanical Strength Test

To assess the scaffolds’ capacity to withstand force, a simple compression test was performed (compressive load). This study was adapted by Salleh et al., 2022 [41]. Three N is the total load placed on the constructed scaffolds. The following formula was used to calculate the compression of modulus:

Compression (%) = Ai − Af/Ai × 100

where Ai is the area of thickness before compression, and Af is the area of thickness after compression.

The resilience of the scaffolds, or their capability to keep their original form despite pressure, was taken from Salleh et al., 2022 [39]. The biocomposite scaffolds were subjected to a total weight of 3 N of metal for a duration of 5 min. After that, the bioscaffolds spent 5 min submerged in distilled water. The thickness area of the bioscaffold before compression, during compression and after rehydration was measured using ImageJ software (NIH, Bethesda, MD, USA). The following formula was used to determine the resilience (R):

Resilience (%) = Ai − Ac/Af × 100

where Ai is the area of thickness before compression, Af is the area of thickness after rehydration and Ac is the area of thickness after compression.

### 2.8. Contact Angle

The contact angle was achieved by preparing a biocomposite solution on a glass slide. The biocomposite solution was smeared on top of the glass slide and left to dry. Around 5 µL of distilled water was dropped on the slides, and the contact angle of each water droplets was recorded. ImageJ software (NIH, Bethesda, MD, USA) was used to analyse the results.

### 2.9. Microporous Structure Study

The microstructure of composite bioscaffolds was observed using scanning electron microscopy (SEM) at 15 kV. The composite bioscaffold is fixed with 3% glutaraldehyde and underwent serial dehydration by using ethanol. The scaffolds were pre-frozen and freeze-dried overnight, followed by nanogold-coating for SEM observation. Using measurement software, the scaffolds’ pore sizes were randomly measured. The fibrous structure was examined using a field emission SEM at a greater magnification. Using ethanol, the liquid displacement method of the porosity test was carried out. The data were analysed using the formula below:

Porosity = [(Wf − Wi)/ρV] × 100,

where Wf is final weight, Wi is initial weight, ρ is the density of ethanol and V is the volume of the scaffold.

### 2.10. Energy Dispersive X-ray (EDX)

The contents of the element on the surface of composite bioscaffolds were measured by using energy dispersive X-ray microanalysis (EDX) (Phenom, Eindhoven, The Netherlands). The control group used is an NCL sponge.

### 2.11. Fourier Transform Infrared X-ray (FTIR)

Fourier transform infrared (FTIR) spectroscopy was performed to determine the functional groups present in the bioscaffold (PerkinElmer, Waltham, MA, USA). The FTIR spectra have been obtained from a small part of composite flakes using the FTIR spectrophotometer. A wavelength range of 4000–500 cm^−1^, at 2 cm^−1^ resolution of per point at room temperature, was conducted and measured.

### 2.12. X-ray Diffraction (XRD)

The XRD analysis of bioscaffolds was conducted by using a Bruker D8 Advance X-ray diffractometer equipment (Bruker AXS GmbH, Karlsruhe, Germany). At a voltage of 40 kV with a broad focus Cu tube running supplied the samples for measurement with a current of 25 mA at a wavelength of 0.154 nm. The information was gathered based on 2-theta versus the intensity (a.u.) chart.

### 2.13. Cytotoxicity of Elastin towards Human Dermal Fibroblasts

Elastin dose response and the cytotoxicity towards human dermal fibroblasts (HDF) cells were evaluated using MTT assay kit (3-(4,5-dimethylthiazol-2-yl)-2,5-diphenyltetrazolium bromide). The cells were seeded in a well-plate and fed with nutrient medium for 1 day. The cells were then exposed to elastin containing a medium at different concentrations for 24 h. Then, cells treated with the medium containing elastin were then reacted with MTT and incubated at 4 h at 37°C and then solubilized with dimethylsulfoxide (DMSO). The absorbance reading was then measured at 540 nm.

### 2.14. Elastin Assay

An elastin assay can be performed by using an Elastin Assay Kit (Fastin^TM^, Biocolor Ltd., Belfast, Northern Ireland, UK) in which the amount of elastin being released from the scaffold can be measured. The scaffold was first degraded by soaking the scaffold in DPBS at 37 °C at different time points. The DPBS containing degraded contents of the scaffold was then collected and underwent the elastin assay by using the Fastin^TM^ Elastin Assay kit. The kit contains a few reagents and steps which involve elastin isolation, recovery of the elastin–dye complex, release of the elastin bound dye and, lastly, the elastin measurement, which is at 513 nm.

### 2.15. Statistical Analysis

GraphPad Prism 8.0 (GraphPad Software, Inc., San Diego, CA, USA) was used to analyse the data gathered. The information wasdisplayed as mean ± standard deviation (SD) gathered from all characterization parameters. In both one-way and two-way analyses, an ANOVA was used to compare treatment groups with the control group (NCL Sponge). The difference in the data was considered significant with a *p*-value less than 0.05. All quantitative data values were evaluated based on three (n = 3) replicate experiments.

## 3. Results

### 3.1. Gross Appearance

**Figure 3:**
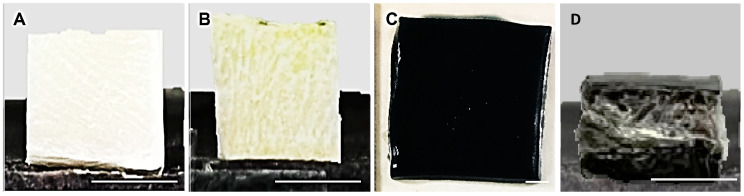
Gross appearance of the scaffolds from each group. (**A**) NCL Sponge, (**B**) CL Sponge, (**C**) hybrid film and (**D**) CL/CL bilayer. The scale indicates 0.5 cm.

Figure 3 represents the gross appearance of the groups fabricated for this study. Based on the figure, the NCL sponge appears to be white in colour as it is the original colour of the collagen from OTC-I. The CL sponge appears to be yellowish in colour as it was crosslinked with genipin. The blue colour from the reaction of genipin with the polymer did not usually appear, because it was newly fabricated and not exposed to the air or surroundings. Next, the hybrid film and CL/CL bilayer appears to be blue in colour due to the bioscaffolds being crosslinked with genipin.

### 3.2. Physicochemical and Mechanical Analysis and SEM of the Scaffolds

**Figure 4:**
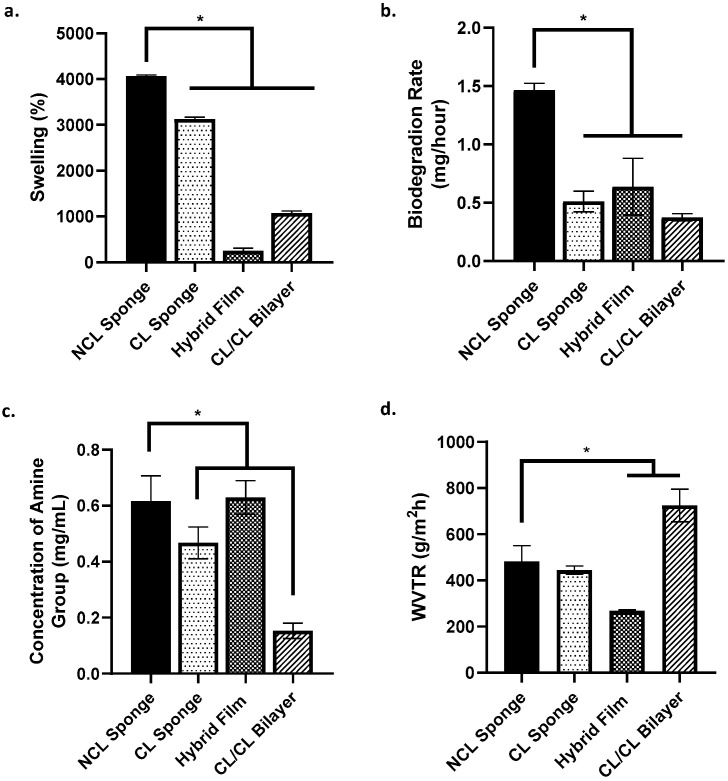
Physicochemical analysis of the scaffolds. (**a.**) % of swelling ratio, (**b.**) rate of biodegradation, (**c.**) concentration of amine group and (**d.**) water vapour transmission rate. * indicates that *p* < 0.0001.

The physicochemical properties of the bioscaffolds fabricated were evaluated based on swelling studies, in vitro biodegradation, crosslinking degree, water vapour transmission rate, simple mechanical study and porosity. Based on Figure 4a, all groups, except for hybrid film, showed a swelling ratio of more than 1000% (acceptable ratio for good swelling properties). The NCL sponge demonstrated the highest swelling percentage compared to other groups. The biodegradation rate of each group was evaluated by the enzymatic degradation approach based on Figure 4b. The rate of NCL sponge biodegradation was higher than the other groups, which was 1.46 ± 0.06 mg/h. It shows that the NCL sponge was able to degrade faster than other groups due to it not being crosslinked. Next, the level of crosslinking was evaluated by measuring the free amine group using the ninhydrin test (Figure 4c). Comparing crosslinked and non-crosslinked bioscaffolds, most of them showed higher free amine concentrations, except for the CL/CL bilayer, which was only 0.153 ± 0.03 mg/mL. This indicates a higher crosslinking degree in the CL/CL bilayer due to the crosslinking of genipin, collagen and the film containing gelatin, PVA and elastin together, leaving no free functional groups to be detected. WVTR aims to look at the measure of the passage of water vapour, gaseousness and its permeability. Based on Figure 4d, the water vapour transmission rate presented the highest for the CL/CL bilayer at 724.65 ± 70.69% due to the scaffold being double layered, providing more barrier for the water vapour to pass through.

**Figure 5:**
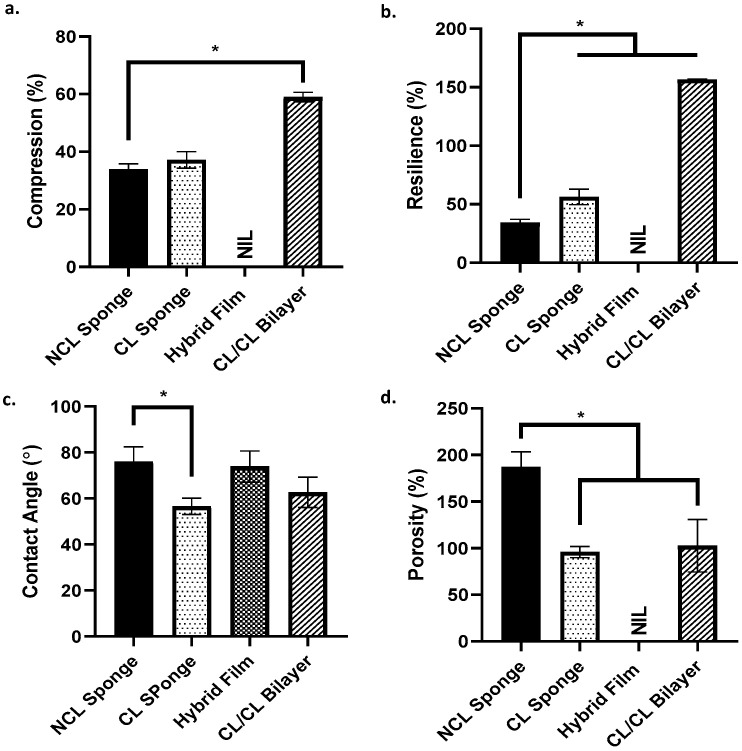
The characterization of the scaffold. (**a.**) Compression, (**b.**) resilience, (**c.**) contact angle and (**d.**) porosity. * indicates that *p* < 0.0001.

Simple mechanical studies were completed by looking at the ability of the scaffold to withstand pressure and the scaffold’s capability to regain its original shape after being compressed. Based on Figure 5a,b, as for the results for compression (%), the CL/CL bilayer group shows the highest percentage of the scaffold to withstand the pressure (58.99 ± 1.16%) compared to other groups. The hybrid film group was stated as NIL because the film is too thin and is not suitable for the study. As for resilience (%), the CL/CL bilayer group again showed the highest percentage (156.53 ± 0.1%) compared to the other groups. It shows that the scaffold can retain its original shape after being compressed. The NCL sponge showed the lowest percentage, proving that it cannot retain its original shape and size even after being compressed. The wettability of the surface of the scaffold could be determined based on a contact angle study. As shown in Figure 5c, all groups exhibited a contact angle less than 90°, thus showing that all groups have a good surface wettability. The porosity of the scaffold is shown in Figure 5d. The NCL sponge group showed a higher percentage of porosity than other groups due to the scaffold not being crosslinked with genipin. Crosslinked groups, CL sponge and CL/CL bilayer showed a lower percentage of porosity; however, they are still >50%. The hybrid film was labelled NIL as it is unsuitable for this study.

**Figure 6:**
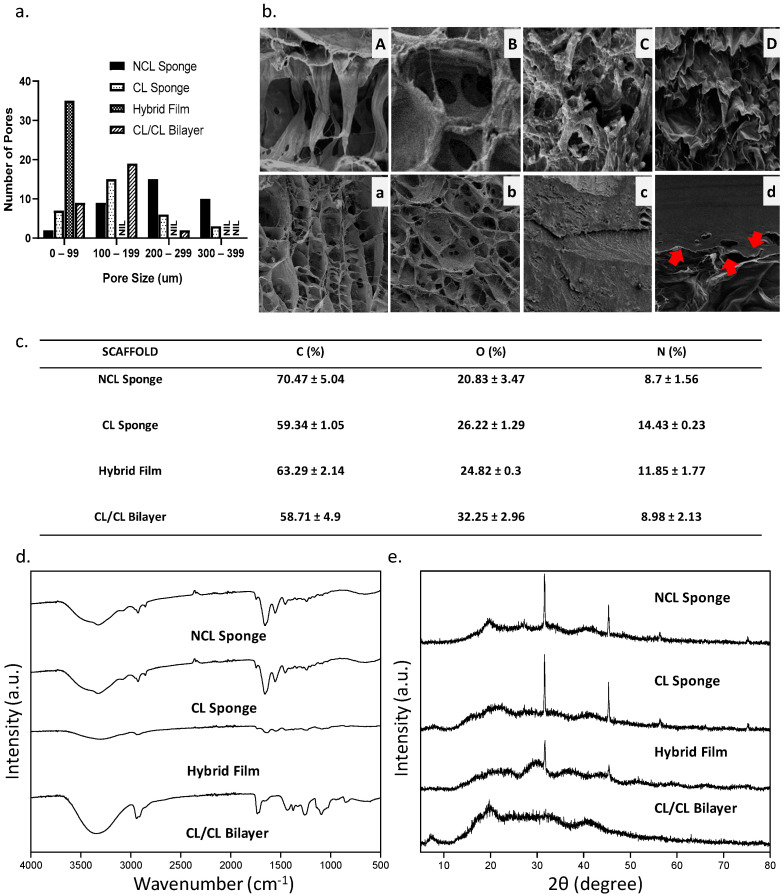
(**a.**) The pore sizes of the scaffold, (**b.**) the SEM images of the scaffold where (**A**) and (**a**) is NCL Sponge, (**B**) and (**b**) is CL Sponge, (**C**) and (**c**) is Hybrid Film and (**D**) and (**d**) is CL/CL Bilayer (**A**–**D**: 500× magnification, **C**: 2000× magnification, **a**–**d**: 50× magnification) and the chemical characteristics of the scaffold: (**c.**) EDX results, (**d.**) FTIR results and (**e.**) XRD results.

Figure 6a showed the pore size of each scaffold in each group. In the range 0–99 µm, the hybrid film shows the highest number of pore size range. In the range 100–199 µm, the CL/CL bilayer unravelled the highest number of the pore size range. Next, in the range 200–299 µm, the NCL sponge showed the highest pore size range. Lastly, the range 300–399 µm demonstrated only 2 groups: NCL sponge and CL sponge. The red arrows in Figure 6b labelled D indicate the separation of the bilayer scaffold between the top layer (hybrid film) and the bottom layer (collagen sponge).

### 3.3. Chemical Characterizations

Since the primary components of collagen and elastin are carbon, oxygen and nitrogen, the compositional analysis provided by the EDX was utilized to determine whether the bioscaffold was homogeneous. The data of EDX can be observed in Figure 6c, where the percentages of carbon (C), oxygen (O) and nitrogen (N) in the fabricated bioscaffolds were not changed significantly. The maximum carbon content was seen in NCL sponge (70.47 ± 5.04%), compared to other groups. The oxygen content for NCL sponge showed the lowest percentage (20.84 ± 3.47%) compared to other groups due to the non-crosslinked bioscaffold. Figure 6d exhibits the FTIR spectra of the bioscaffolds. Similar absorbance was observed in the FTIR spectra of the cross-linked and non-crosslinked groups, with the Amide A in between 3318 and 3298 cm^−1^, Amide I in between 1632 and 1628 cm^−1^, Amide II in between 1551 and 1545 cm^−1^ and Amide III in between 1285 and 1238 cm^−1^ being the most similar [41]. In the control group, NCL sponge described the properties of amide A due to the NH stretching, amide B resulted to CH_2_ asymmetrical stretching, amine I resulted to NH bending, amine II due to CN stretching and amine III. Due to the mixture of many biomaterials, every group showed the presence of each functional group similar to the NCL sponge. Lastly, a material’s crystallographic structure can be evaluated via XRD. All groups exhibited almost similar diffractogram patterns based on Figure 6e. All groups were in the amorphous phase based on the analysis. The NCL sponge, CL sponge, hybrid film and CL/CL bilayer had high percentages of amorphous phases, which were 63.7%, 78.8%, 62.8% and 69.2%, respectively.

### 3.4. Cytotoxicity Effect of Elastin and Elastin Assay

**Figure 7:**
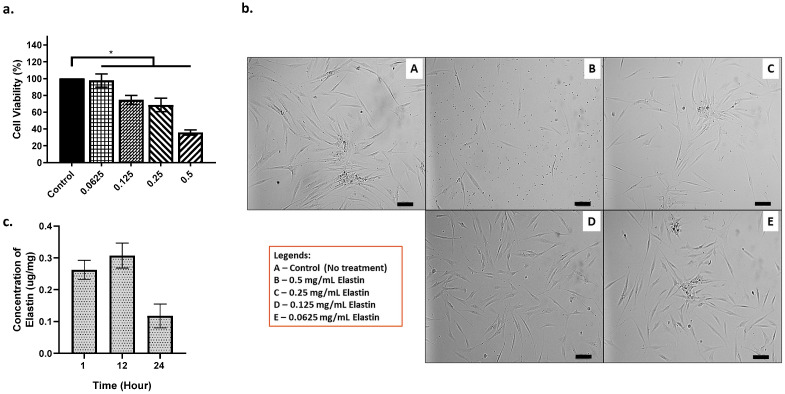
(**a.**) The HDF cells interaction towards elastin (cell viability %), provided with (**b.**) the images of HDF at different concentrations of elastin (100 μm) and (**c.**) the concentration of elastin being released. * indicates that *p* < 0.0001.

The cytotoxicity of elastin was determined by using the MTT assay method. Based on Figure 7a,b, the lower the concentration of elastin, the better the cell viability (%). Starting from 0.5 mg/mL of elastin, the cell viability is lower than 60%. The 0.25 mg/mL is the final concentration with high potential and was chosen as the best concentration for the elastin’s maximum potential. Next, the concentration of elastin being released from the scaffold after biodegradation can be observed in Figure 7c. After 12 h, the elastin concentration showed the highest at 0.3 (μg/mg). The concentration of elastin being released decreased after 24 h.

## 4. Discussion

The main concern of fabricating a scaffold from biomaterials will always be the capabilities of a scaffold to mimic human nature, especially for a scaffold and a specific target application in tissue engineering and medical applications. In this study, an alternative to the gold standard of wound healing treatment, or other known medical treatment, was fabricated to solve the current issues or help improve the field of medical applications. Therefore, a list of parameters needs to be completed by characterizing the physicochemical and mechanical properties in search of the best-fabricated scaffold that may help in future regenerative medicine field and tissue engineering applications. 

Due to their unique qualities and limitations, one-layer wound dressings cannot satisfy all therapeutic requirements. Bilayer wound dressings, consisting of two layers with distinct qualities, have attracted much attention [42,43,44]. In this study, a bilayer scaffold was fabricated successfully from many different sources, such as collagen from the ovine tendon collagen type I (15 mg/mL) as a sponge and hybrid film from a mixture of gelatin (4% *w*/*v*), PVA (4% *w*/*v*) and elastin (0.25 mg/mL), forming an acellular heterogeneous porous scaffold. The novelty of this study is to fabricate an acellular scaffold that was degraded from time to time with the help of genipin (0.1% *w*/*v*), a crosslinker, as well as having elastin as the bioactive that potentially helps in reducing over-contraction during wound healing. A combination of collagen as a sponge that can mimic the extracellular matrix of the human body and gelatin as another layer (film) mixed with PVA to support mechanically helps release the elastin and act as an antibacterial barrier with antibacterial properties in future studies. 

Looking at its characteristics, an acellular scaffold requires cells to migrate into the scaffold to help in cell proliferation and remodelling during the wound healing phase. Thus, the scaffold needs to be degraded post-implantation. Luckily, the biomaterials involved in the making of this scaffold are biodegradable. The CL/CL bilayer demonstrated a slow biodegradation rate at about 0.37 ± 0.03 mg/h in a mimic environment, such as the human body. The rate of biodegradation is perfect for wound healing, as generally, the chosen biomaterials in wound healing should last at least 2 weeks (14 days), and the implanted site may regenerate the newly produced tissue during this time [33,45]. With the help of genipin, it helps control the biodegradation time by stabilizing the scaffold by forming covalent bondings [46]. The crosslinker also affects the swelling capability of the scaffold. Without the crosslinker, the swelling (%) could be higher than the normal range due to its high porosity. The CL/CL bilayer group showed a swelling percentage of 1078.734 ± 42.33%, within the optimum range of swelling. The fabricated bilayer scaffold can be employed as wound dressings for deep wounds by absorbing excess exudates even from significant exudate amounts to the achieved swelling index [41]. Over-absorption might cause drying in the wounded area and slow the healing process. A scab will develop in a too-dry wound bed, preventing healing and wound contraction. The surrounding tissue and underlying collagen matrix will dry out at the wound edge. However, excessive exudate from a wound can cause maceration and excoriation, where the wound bed becomes saturated and moist around the site of the wound [47,48]. A wound that is too dry can hurt the patient, while a wound that is too wet can result in saturated clothing, making the patient uncomfortable and upset. Creating an environment at the wound bed that optimizes the healing process is a problem for physicians. Thus, it is important to measure water absorption ability as it absorbs excess exudates and maintains the microenvironment [49]. The crosslinking degree of the desired scaffold can be measured by looking at free amine groups. A highly crosslinked scaffold will release a lesser amine group due to the functional groups forming covalent bonding, thus the desired scaffold, CL/CL bilayer, showed a good crosslinking degree. Based on Nike et al. [36], the development of heterocyclic amino linkage crosslinking between genipin and the amino group is caused by a nucleophilic attack of the gelatin’s amino group with the C-3 olefinic carbon atom of the genipin, triggered by the opening of the dihydropyran ring.

The rate of water vapour or gaseous transfer from the inner side of the body towards the outside of the body through the scaffold is also important to came across. The permeability of the scaffold to allow water vapour or gaseous exchange is important to provide moist environment on the wound bed. The CL/CL bilayer is within the range of the optimum water vapour permeability, compared to the known commercially available skin substitutes [50]. Next, the bilayer scaffold showed a strong mechanical structure with good compression and resilience (%) properties. The mechanical structure needs to be strong enough to avoid the scaffold becoming crumpled or collapsing due to bad handling during storage or even during the handling for medical applications. Other than that, surface hydrophilicity of a scaffold is important for the attachment of the cells (cell adhesion) [51]. The CL/CL bilayer provides a good surface wettability at an angle below 90°; thus, the hydrophilic surface may promote cell attachment. Both collagen and gelatin improve the hydrophilicity of the surface [37,51].

Other than that, a scaffold needs to be porous for the cells to migrate, especially during wound healing phases. Based on the SEM images, the distribution of pores has some variety of sizes. However, the CL/CL bilayer showed most of its pore size in the range of 100–200 µm, which indicates a good pore size and is recommended for skin wound healing [52,53,54]. To support this statement, the porosity study was also conducted with ethanol. Due to its non-polar nature and lack of interaction with polymeric fibres, ethanol easily permeates the scaffold and fills all the sample’s holes, giving rise to the total volume of pores [55]. The CL/CL bilayer showed a lower percentage of porosity, supporting the statements above. The EDX data shown conclude that all bioscaffolds including CL/CL bilayer are homogenous. The FTIR analysis showed the common absorbance peaks for amines I, II and III, which indicates that collagen and gelatin together with elastin have similar functional groups; thus, the functional groups mentioned appeared in the peaks. The CL/CL bilayer is also amorphous, based on the peak in XRD result. All the physicochemical and mechanical findings further support that the fabricated bioscaffolds mimic the microstructure of natural skin tissue while maintaining their original native characteristics.

Lastly, in addition to providing mechanical elasticity, elastin promotes skin regeneration and eventually reduces wound contraction by working on cells to aid in wound healing [56]. The chosen concentration of elastin is 0.25 mg/mL as it showed the best concentration that maximizes the potential of elastin towards human dermal fibroblasts, as well as the wound healing application. Although it has a lower cell viability compared to other lower concentrations of elastin, it still shows a good cell viability, which is above 60%. The elastin assay was completed to look at the release of the elastin from the scaffold. It is important to know the concentration of elastin released by the scaffold as there might be some unintentional bonding between genipin, collagen, gelatin and elastin. Based on the result, it is the main priority to know that the elastin was released from the scaffold. However, further evaluation needs to be performed to look at its functionality after being released, if there are any changes on the functionality of the soluble protein (elastin).

## 5. Conclusions

To conclude, the CL/CL bilayer scaffold has been successfully fabricated through the methods of freeze-drying and the solution casting method with the help of a crosslinker. The mixture of natural and synthetic materials should be further explored as it shows promising findings. The bilayer showed a promising finding in terms of its physicochemical and mechanical properties for the application of an alternative skin substitute. Not only could it help in improving the skin’s wound healing but, with the addition of elastin, it could help in reducing over contraction that may cause scars. Most importantly, further biocompatibility studies could be completed to look at the cell–scaffold interaction. The potential of the CL/CL bilayer being used in the future and commercially available at an affordable cost will be anticipated, especially in the tissue engineering and regenerative medicine field.

## Data Availability

The data presented in this study are available on request from the corresponding author.
